# Sustainable microbial cell nanofactory for zinc oxide nanoparticles production by zinc-tolerant probiotic *Lactobacillus plantarum* strain TA4

**DOI:** 10.1186/s12934-020-1279-6

**Published:** 2020-01-15

**Authors:** Hidayat Mohd Yusof, Rosfarizan Mohamad, Uswatun Hasanah Zaidan, Nor’Aini Abdul Rahman

**Affiliations:** 1 Department of Bioprocess Technology, Faculty of Biotechnology and Biomolecular Sciences, Universiti Putra Malaysia, 43400 Serdang, Selangor Malaysia; 2 Department of Biochemistry, Faculty of Biotechnology and Biomolecular Sciences, Universiti Putra Malaysia, 43400 Serdang, Selangor Malaysia; 3 Bioprocessing and Biomanufacturing Research Centre, Faculty of Biotechnology and Biomolecular Sciences, Universiti Putra Malaysia, 43400 Serdang, Selangor Malaysia

**Keywords:** Biological synthesis, Extracellular, FT-IR, Lactic acid bacteria, *Lactobacillus plantarum*, Probiotic, Zinc-tolerance, Zinc oxide nanoparticles

## Abstract

**Background:**

The use of microorganisms in the biosynthesis of zinc oxide nanoparticles (ZnO NPs) has recently emerged as an alternative to chemical and physical methods due to its low-cost and eco-friendly method. Several lactic acid bacteria (LAB) have developed mechanisms in tolerating Zn^2+^ through prevention against their toxicity and the production of ZnO NPs. The LAB’s main resistance mechanism to Zn^2+^ is highly depended on the microorganisms’ ability to interact with Zn^2+^ either through biosorption or bioaccumulation processes. Besides the inadequate studies conducted on biosynthesis with the use of zinc-tolerant probiotics, the understanding regarding the mechanism involved in this process is not clear. Therefore, this study determines the features of probiotic LAB strain TA4 related to its resistance to Zn^2+^. It also attempts to illustrate its potential in creating a sustainable microbial cell nanofactory of ZnO NPs.

**Results:**

A zinc-tolerant probiotic strain TA4, which was isolated from local fermented food, was selected based on the principal component analysis (PCA) with the highest score of probiotic attributes. Based on the 16S rRNA gene analysis, this strain was identified as *Lactobacillus plantarum* strain TA4, indicating its high resistance to Zn^2+^ at a maximum tolerable concentration (MTC) value of 500 mM and its capability of producing ZnO NPs. The UV–visible spectroscopy analysis proved the formations of ZnO NPs through the notable absorption peak at 380 nm. It was also found from the dynamic light scattering (DLS) analysis that the Z-average particle size amounted to 124.2 nm with monodisperse ZnO NPs. Studies on scanning electron microscope (SEM), energy-dispersive X-ray (EDX) spectroscopy, and Fourier-transform infrared spectroscopy (FT-IR) revealed that the main mechanisms in ZnO NPs biosynthesis were facilitated by the Zn^2+^ biosorption ability through the functional groups present on the cell surface of strain TA4.

**Conclusions:**

The strong ability of zinc-tolerant probiotic of *L. plantarum* strain TA4 to tolerate high Zn^2+^ concentration and to produce ZnO NPs highlights the unique properties of these bacteria as a natural microbial cell nanofactory for a more sustainable and eco-friendly practice of ZnO NPs biosynthesis.

## Background

Zinc oxide nanoparticles (ZnO NPs) have gained worldwide attention as multifunctional nanomaterials due to their distinctive properties of being versatile semiconductor and piezoelectric properties [1], which are different than their bulkier counterparts. Recently, ZnO NPs are utilized in various applications, such as pharmaceuticals [2], cosmetics [3], photocatalysis [4], and dietary supplement for animals [5]. Many chemical methods are proposed for the synthesis of ZnO NPs, including sol–gel process, solvent evaporation and precipitation from microemulsion [6]. However, the methods are not environmentally-friendly due to the use of harsh chemicals for reduction and stabilizing processes, which will bind to the ZnO NPs, and limit their biological application [5]. Moreover, these process also contribute to secondary pollution by generating toxic by-products. Hence, the development of more sustainable approaches in preparing ZnO NPs are emphasized and in-depth studies are conducted to replace the conventional methods.

Recently, microbial synthesis of metal nanoparticles (NPs) is widely employed due to the low cost, biocompatibility, and being eco-friendly [5]. A number of microorganisms, including bacteria, fungi and yeast are investigated due to their efficiency in Zn^2+^ sorption and ZnO NPs synthesis. Bacteria such as *Lactobacillus* produce ZnO NPs intracellularly [7]. Meanwhile, fungi and yeast such as *Aspergillus aeneus* and *Pichia fermentans*, respectively, produce ZnO NPs extracellularly [8, 9]. Among the microorganisms, lactic acid bacteria (LAB) receive significant attention due to their safety in handling and food-grade status, which are “generally recognized as safe” (GRAS) in food production and preservation. In addition, some LAB strains exhibit probiotic properties for human and animal consumption, contributing to health-promoting properties [10].

Furthermore, LAB efficacy in mediating the synthesis of ZnO NPs was observed in several studies [6, 9–12]. It was reported that LAB can produce selenium [13], gold [14], and silver NPs [15]. Overall, LAB is a useful cell factory for the formation of NPs due to these features. As Gram-positive bacteria, LAB have a thick cell wall consisting of peptidoglycan, lipoteichoic acid, protein, and polysaccharides [16]. The layers function as the sites for the biosorption and bioreduction of metal ions, due to their negative electrokinetic potential features, which attract the metal cations to initiate NPs biosynthesis [7]. Former study by Król et al. [7] proposed that the deprotonated carboxyl groups (functional group) present on the cell surface of *L. paracasei* strain interact with the zinc-aqua complexes (electron acceptor) to form ZnO NPs. Also, LAB produce a wide range of exopolysaccharides (EPS) [17], which serve as the additional binding sites for the cationic metal ions and protect the microorganisms from toxic metal stresses [18].

**Fig. 1:**
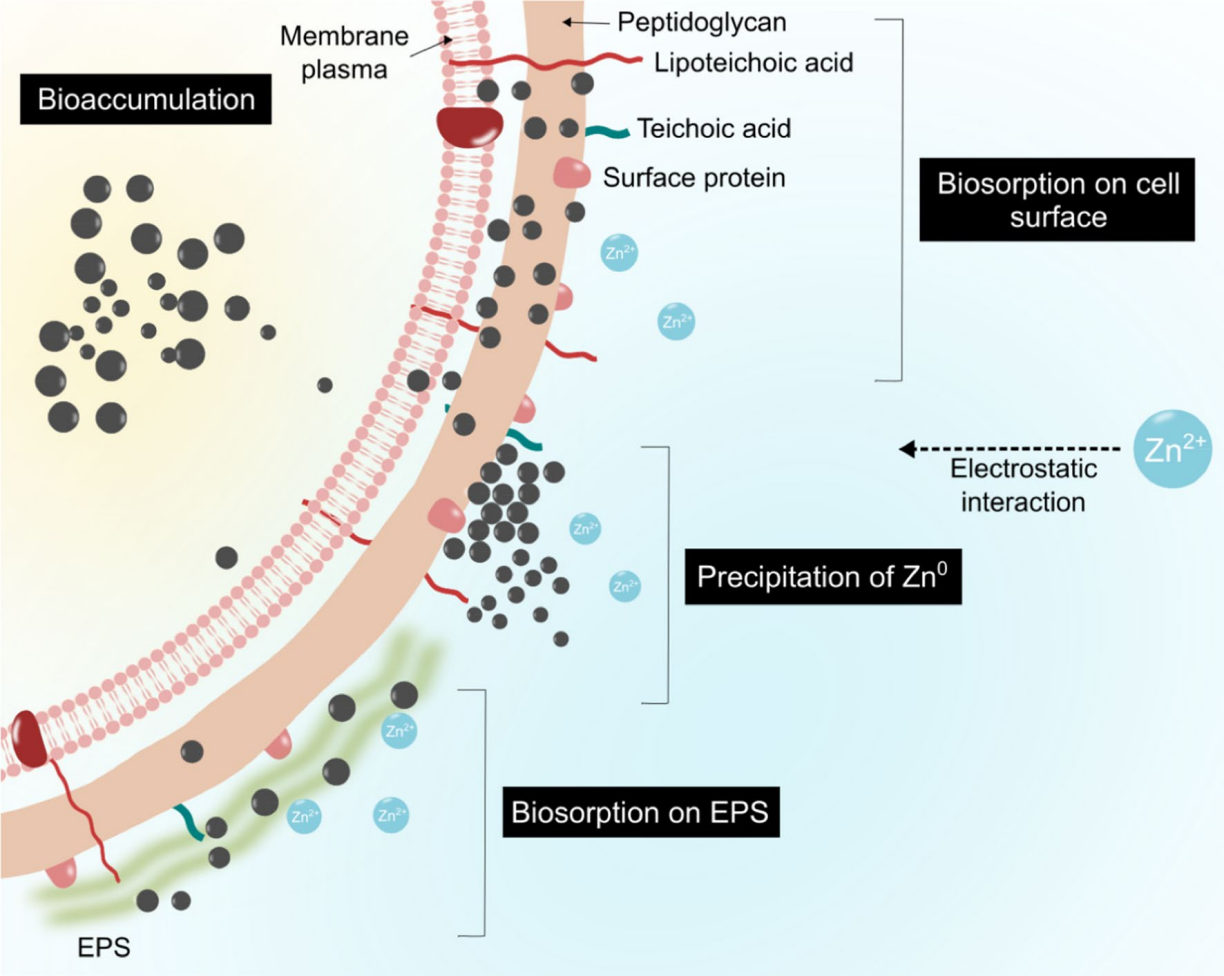
Proposed mechanisms of bacteria in resisting metal ion and produce NPs simultaneously. Biosorption occurs on the bacterial cell wall, which involves the binding of metal cations to the negatively charged functional groups on the bacteria such as carboxyl, phosphate, and hydroxyl [22]. EPS secreted by bacteria also act as biosorption site in the form of biofilm, to tolerate metal ions by trapping them within the EPS matrix and reducing them to the less toxic metal [22]. Precipitation is one of the mechanisms that lower the metal ion toxicity through the reaction between anions such as hydroxyl ion and metal ions (cation) that occurs either intra- or extracellularly. Unlike biosorption, bioaccumulation is an active metabolic process that requires energy and intracellular metal ions binding, with the help of low molecular weight proteins such as metallothioneins to facilitate the process [22, 26]

It is suggested that the biosynthesis of metal or metal oxide NPs is influenced by the microorganisms’ ability to tolerate with metal ion. In fact, high metal stresses affect microbial activity. Under this condition, the microorganisms will interact with metal ions and reduce them to nanoscale metal particles [17, 18]. This interaction enables microorganisms to act as microbial nanofactory. Bacteria adopt several strategies to encounter the toxicity of metal ions via metal ion biosorption and bioaccumulation. Biosorption is a passive and non-metabolically mediated process that involves binding, ion exchange, chelation and precipitation, which depend on the presence of functional groups on the bacterial cell walls [19–21]. This is followed by bioaccumulation, in which the metal ions will enter into the cell and react with bacterial intracellular structures [20, 22]. Figure 1 proposed the mechanisms of Gram-positive bacteria in resisting metal ion and reducing it to the respective metal NPs.

A number of metal-tolerant microorganisms have been isolated from various ecological niches [7, 23] to recover metal NPs. However, the studies conducted on zinc-tolerant probiotic in the biosynthesis of ZnO NPs are limited. Over the last decades, there is heavy usage of probiotic due to its ability to tolerate and bind to various metal ions such as copper (Cu) [24], arsenic (As) [25], zinc (Zn) [19], cadmium (Cd) [21, 22], selenium (Se), and lead (Pb) [26, 27] for toxicological and environmental purposes. However, there has been no proper explanation regarding the mechanisms in which this microorganism resists to Zn^2+^ and produces ZnO NPs simultaneously. Furthermore, the use of probiotic LAB contributes to many benefits, due to the non-pathogenic property, simplicity, and abundance in food products. The metal-tolerant LAB-based probiotic can be the food-grade ZnO NPs producer and adopted as dietary strategy. This is applicable in heavy metal decontamination in the gastrointestinal tract (GIT) of humans and animals, which are exposed to high metal content.

Putting the high number of LAB structures and metabolic features into consideration, diverse outcomes could be predicted in the mechanisms of NPs formation. Therefore, this study aims to investigate the potential of zinc-tolerant LAB equipped with probiotic properties to be utilized as microbial nanofactory for more sustainable productions of ZnO NPs. In this study, the zinc-tolerant probiotic was isolated from locally fermented food, which is capable to resist high Zn^2+^ concentration and produce ZnO NPs. The biosynthesized ZnO NPs were validated and characterized by UV–visible spectroscopy and dynamic light scattering (DLS). The underlying mechanisms of ZnO NPs formation were scrutinized using scanning electron microscope (SEM), energy-dispersive X-ray (EDX) spectroscopy, and Fourier-transform infrared spectroscopy (FT-IR).

## Results

### Isolation and morphological characterization

A total of 125 LAB strains from different sources were obtained. All strains were Gram-positive cocci and bacilli with the catalase-negative trait. All the strains were used for zinc tolerance pre-screening. Eighteen (14.4%) strains were able to grow in the presence of 10 mM Zn^2+^. All of the potential strains were Gram-positive and rod-shaped. The colony morphologies of the strains were whitish cream and circular and the strains were identified as *Lactobacillus*.

### The maximum tolerable concentration (MTC) of LAB strains

**Fig. 2:**
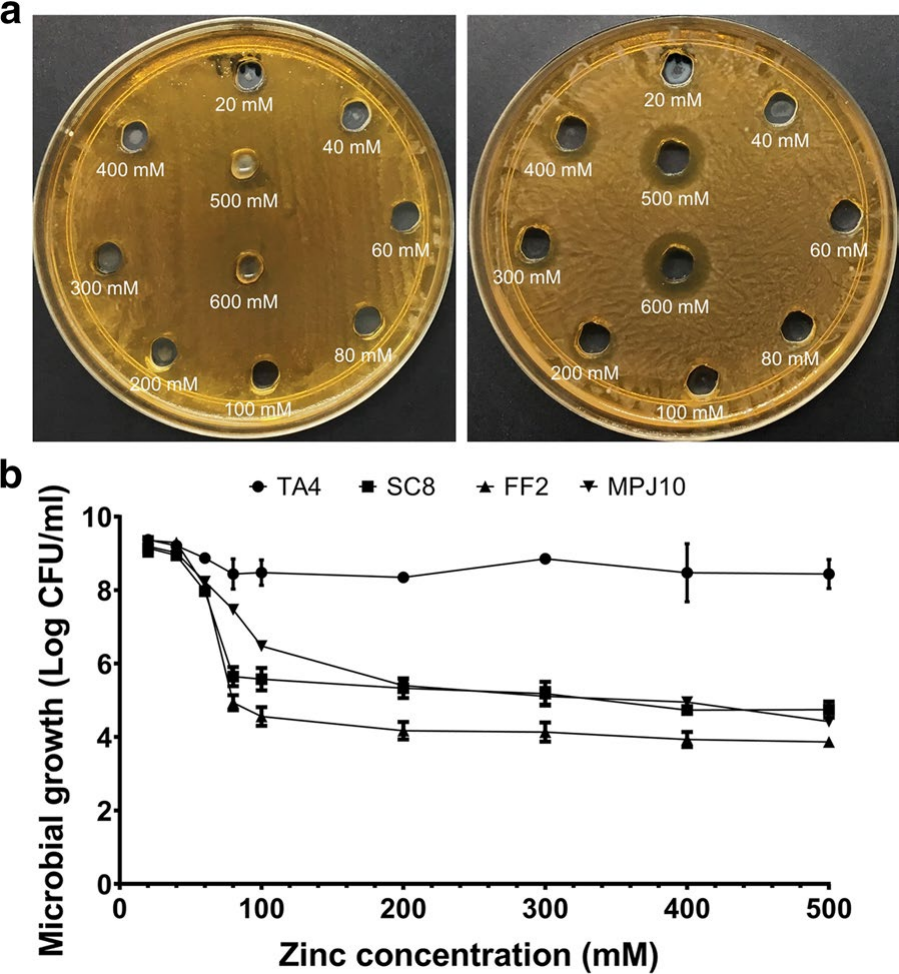
Agar well diffusion method to determine the MTC of LAB strains against various Zn^2+^ concentrations **a** zinc-tolerant strain [Left], less zinc-tolerant strain [Right]. Microbial growth of LAB strains in various Zn^2+^ concentrations by using tube dilution method (**b**). Data are expressed as mean (*n *= 3) ± SD

The maximum tolerable concentration (MTC) values of the LAB strains are presented in Table 1. The MTC of the 18 LAB strains were investigated to determine the ability of the bacteria to tolerate various concentrations of Zn^2+^. Out of 18 LAB strains, four strains (TA4, SC8, FF2, and MPJ10) exhibited the highest MTC values at 500 mM. Figure 2a depicts the MTC assay using agar well diffusion method, which showed different levels of tolerance by the LAB strains against various Zn^2+^ concentrations. Nevertheless, the agar well diffusion method was unable to determine the viability of the surviving cell; hence, the tube dilution method was performed to measure the number of propagating LAB in different Zn^2+^ concentrations. Based on the presented results in Fig. 2b, the ability of each strain was varied in various concentrations and interestingly, strain TA4 demonstrated a remarkable growth at the highest concentration of Zn^2+^, which was only a onefold reduction of growth (log CFU/mL). Meanwhile, other strains showed up to four- to fivefold reduction of growth with increasing Zn^2+^ concentration, starting from a concentration of 80 mM. However, at those particular Zn^2+^ concentrations, the strains were still able to propagate and survive, which indicate their tolerance against Zn^2+^. Therefore, strains TA4, SC8, FF2, and MPJ10 were selected for further screening of their probiotic attributes.

**Table 1:** Zinc tolerance of the LAB strains against different zinc concentrations

Strain code	Inhibition zone (mm)									
	Zn^2+^ concentration (mM)									
	20	40	60	80	100	200	300	400	500	600
TA4^a^	R(–)	R(–)	R(–)	R(–)	R(–)	R(–)	R(–)	R(0.5)	R(1)	S(1.2)
TP2	R(–)	R(–)	R(–)	R(–)	R(0.5)	S(2)	S(3)	S(4)	S(4.5)	S(6)
TP6	R(–)	R(–)	R(–)	R(–)	R(–)	S(2)	S(4)	S(4)	S(4)	S(5)
SC2	R(–)	R(–)	R(–)	R(1)	R(1)	S(1.5)	S(3)	S(4)	S(4.5)	S(6)
SC4	R(–)	R(0.5)	R(0.5)	R(1)	R(1)	S(2)	S(3)	S(4)	S(4.5)	S(5)
SC7	R(–)	R(–)	R(–)	R(–)	R(–)	R(1)	S(2)	S(2)	S(3)	S(4.5)
SC8^a^	R(–)	R(–)	R(–)	R(–)	R(–)	R(0.5)	R(0.5)	R(1)	R(1)	S(1.5)
MP3	R(–)	R(–)	R(–)	R(0.5)	R(0.5)	S(2)	S(2)	S(2)	S(3)	S(4)
MP6	R(–)	R(–)	R(0.5)	R(1)	R(1)	S(2)	S(4)	S(4.5)	S(4.5)	S(5)
FF2^a^	R(–)	R(–)	R(–)	R(–)	R(–)	R(–)	R(1)	R(1)	R(1)	S(2)
FF4	R(–)	R(–)	R(–)	R(–)	R(–)	S(2)	S(2.5)	S(3)	S(3)	S(4)
MPJ1	R(–)	R(–)	R(–)	R(–)	R(–)	S(2)	S(3)	S(3.5)	S(4)	S(6)
MPJ2	R(–)	R(–)	R(–)	R(–)	R(–)	S(1.5)	S(2)	S(3)	S(5)	S(5)
MPJ4	R(–)	R(–)	R(–)	R(–)	R(–)	S(2)	S(2)	S(3)	S(4)	S(5)
MPJ5	R(–)	R(–)	R(0.5)	R(0.5)	R(1)	S(2)	S(3)	S(5)	S(5)	S(5.5)
MPJ7	R(–)	R(–)	R(–)	R(–)	R(–)	S(2.5)	S(3)	S(4)	S(5)	S(5)
MPJ9	R(–)	R(–)	R(–)	R(–)	R(–)	S(2)	S(2.5)	S(2.5)	S(4)	S(5)
MPJ10^a^	R(–)	R(–)	R(–)	R(–)	R(–)	R(0.5)	R(0.5)	R(1)	R(1)	S(2)

The results of the inhibition zone are shown in parenthesis

*S* sensitive (≥ 1.5 mm), *R* resistant (≤ 1 mm)

^a^Indicating the strains that exhibited the highest MTC value

### In vitro probiotic characterization of zinc-tolerant LAB

#### Acidic pH, bile salts, and phenol tolerance

**Fig. 3:**
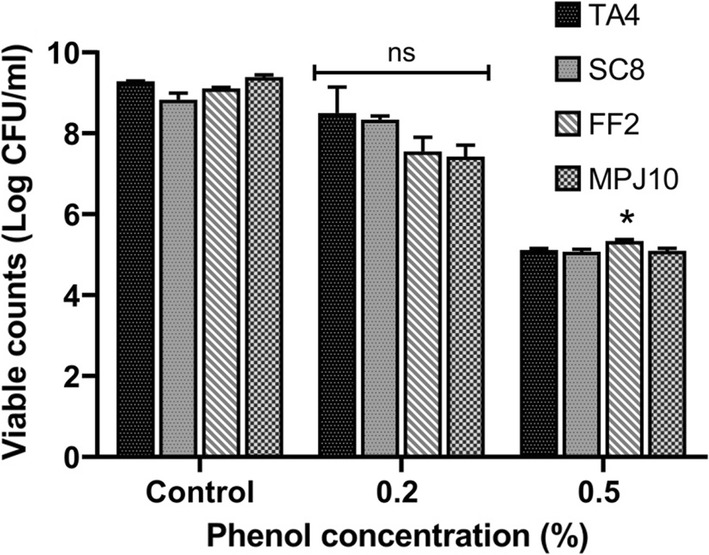
Phenol tolerance of all strains at concentration of 0.2 and 0.5% (v/v). The culture was incubated at 37 C for 24 h. Data are expressed as mean (*n *= 3) ± SD. *p < 0.05. *ns* no significant

Resistance to gastric pH, bile, and phenol are key features for bacteria to be able to survive in the gastrointestinal tract (GIT) and are some of the important selection criteria for probiotic. As shown in Table 2, all the strains survived at pH 2.5 and 3.5. The maximum survival rate percentage was at pH 3.5 ranging from 90.5 to 94.3%, with strain MPJ10 exhibiting the highest value. Meanwhile, at pH 2.5, all the strains showed various survival rates ranging from 59.9 to 85.1%, with strain FF2 exhibiting the highest percentage value and strain SC8 exhibiting the lowest value. However, no significance difference (*p* > 0.05) in survival rate percentage was observed in both tested pH among the strains, which indicate that all strains are able to survive in acidic pH for 4 h. A similar trend was seen in bile tolerance ability, where there was a high survival rate percentage among the strains at bile concentrations of 0.3% and 0.6% ranging from 78.1 to 89.9% and 76.7 to 90.3%, respectively. For phenol tolerance (Fig. 3), all the strains showed the ability to grow under a phenol concentration of 0.2%, whereas at a concentration of 0.5%, the viable counts (log CFU/mL) for all strains decreased.

**Table 2:** Tolerance of the strains to acidic pH and bile salts at 37 °C for 0 and 4 h (log CFU/mL)

Strain	pH						Bile					
	2.5			3.5			0.3%			0.6%		
	0 h	4 h	SR %	0 h	4 h	SR %	0 h	4 h	SR %	0 h	4 h	SR %
TA4	9.22 ± 0.04	9.12 ± 0.01	79^a^	9.36 ± 0.02	9.32 ± 0.06	90.5^a^	8.93 ± 0.07	8.85 ± 0.11	83.2^a^	9.06 ± 0.17	9.01 ± 0.19	88.5^a^
SC8	9.23 ± 0.05	9.00 ± 0.07	59.9^a^	9.24 ± 0.07	9.20 ± 0.10	91.5^a^	9.05 ± 0.18	8.95 ± 0.05	81.5^a^	9.05 ± 0.15	9.00 ± 0.14	90.3^a^
FF2	9.24 ± 0.02	9.16 ± 0.11	85.1^a^	8.98 ± 0.05	8.95 ± 0.08	91.8^a^	9.02 ± 0.02	8.91 ± 0.05	78.1^a^	8.72 ± 0.03	8.67 ± 0.11	88.1^a^
MPJ10	9.35 ± 0.07	9.20 ± 0.11	70.4^a^	9.22 ± 0.05	9.20 ± 0.05	94.3^a^	9.02 ± 0.04	8.97 ± 0.04	89.9^a^	8.75 ± 0.09	8.64 ± 0.04	76.7^a^

The results are expressed as mean (*n *= 3) ± SD, and values within the same column with different superscript letters indicate statistical differences in each strain according to Tukey’s test at *p *< 0.05. The means are presented as log-transformed values of CFU/mL of the strains

*SR%* survival rate percentage

#### Cell surface hydrophobicity, cellular autoaggregation assessment and EPS production

The adhesion characteristics of all the strains is shown in Table 3. Strains TA4 and SC8 demonstrated hydrophobic characteristics with 58% and 56.1% adhering to toluene, which is an apolar solvent. Meanwhile, strains FF2 and MPJ10 showed the lowest percentage indicated their hydrophilic characteristics. For chloroform, strains TA4 and SC8 showed the highest percentage with 54.9% and 54.5%, respectively, whereas strain MPJ10 demonstrated the highest hydrophobic percentage to ethyl acetate with 53.6%. Results for cellular autoaggregation are presented in Table 3. For all the four potential probiotics, the ability to autoaggregate increased by augmenting the incubation time. Among the strains, strain TA4 exhibited the highest value (64%) at 4 h and all strains showed the highest values after 24 h incubation in the range of 64.4–99.3%. For EPS production, none of the strains showed positive results for EPS production.

**Table 3:** Cell surface hydrophobicity activity of LAB strains against different solvents and cellular autoaggregation activity at a different time of incubation and EPS production ability

Strain	Cell surface hydrophobicity (%)			Cellular autoaggregation (%)			EPS production
	Chloroform	Toluene	Ethyl acetate	1 h	4 h	24 h	
TA4	54.93 ± 0.34^a^	57.99 ± 1.04^a^	41.36 ± 1.18^c^	6.14 ± 0.05^ab^	64.00 ± 0.01^a^	99.33 ± 0.03^a^	–
SC8	54.49 ± 0.16^a^	56.05 ± 0.00^a^	44.40 ± 0.10^b^	3.64 ± 0.00^c^	11.93 ± 0.27^c^	88.69 ± 0.09^c^	–
FF2	28.51 ± 3.51^c^	26.87 ± 0.08^b^	35.47 ± 0.41^d^	4.91 ± 0.57^bc^	14.60 ± 0.20^b^	98.03 ± 0.02^b^	–
MPJ10	40.94 ± 0.80^b^	11.41 ± 2.08^c^	53.55 ± 0.64^a^	1.95 ± 0.57^d^	13.62 ± 0.54^b^	64.39 ± 0.19^d^	–

The results are expressed as mean (*n *= 3) ± SD, and values within the same column with different superscript letters indicate statistical differences in each strain according to Tukey’s test at *p *< 0.05

#### Antimicrobial activity

The antagonistic properties of the LAB strains against Gram-negative and Gram-positive pathogens are presented in Table 4. The non-neutralized CFS of all strains were subjected against indicator pathogens *E. coli*, *Salmonella* sp., *S. aureus*, and *S. epidermidis*. Strains TA4, FF2, and MPJ10 demonstrated antagonism against the tested pathogens. Meanwhile, strain SC8 showed high antagonism against *E. coli* and *Salmonella* and least antagonism activity was detected on *S. aureus* and *S. epidermidis*. Moreover, the neutralized CFS of all strains did not show any inhibitory activity against the tested pathogens (data not shown).

**Table 4:** Antibacterial activity of cell-free supernatant (CFS) of the LAB strains

Strain	Final pH of CFS	Zone of inhibition (mm)			
		Gram-negative		Gram-positive	
		*E. coli*	*Salmonella* sp.	*S. aureus*	*S. epidermidis*
TA4	3.41 ± 0.05	19.33 ± 0.58^a^	16.67 ± 1.15^b^	19.00 ± 1.00^a^	17.67 ± 0.58^a^
SC8	3.85 ± 0.14	19.00 ± 1.00^a^	20.00 ± 0.00^a^	13.00 ± 0.00^b^	11.00 ± 0.00^c^
FF2	3.48 ± 0.03	18.33 ± 0.58^a^	15.00 ± 1.00^b^	18.67 ± 0.58^a^	16.00 ± 1.00^ab^
MPJ10	3.36 ± 0.03	18.00 ± 0.00^a^	15.00 ± 0.00^b^	17.67 ± 0.58^a^	15.00 ± 1.00^b^

The results are expressed as mean (*n *= 3) ± SD, and values within the same column with different superscript letters indicate statistical differences in each strain according to Tukey’s test at *p *< 0.05

#### 2,2-Diphenyl-1-picrylhydrazyl (DPPH) free radical scavenging activity (RSA)

**Fig. 4:**
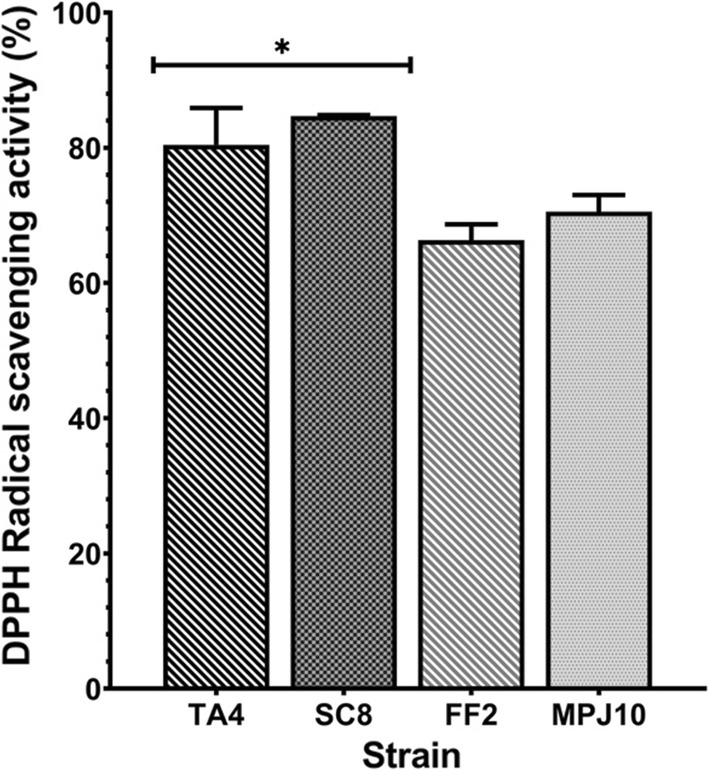
DPPH scavenging activity of the LAB strains. Data are expressed as mean (*n* = 3) ± SD. *p < 0.05

DPPH free radical-scavenging activity of LAB strains is illustrated in Fig. 4. The cell-free supernatant (CFS) of strains SC8 and TA4 exhibited high antioxidant activity at 84.7% and 80.5% (*p* < 0.05), respectively, while strains FF2 and MPJ10 showed the least antioxidant activity with 66.3% and 70.5%, respectively.

#### Principal component analysis (PCA)

**Fig. 5:**
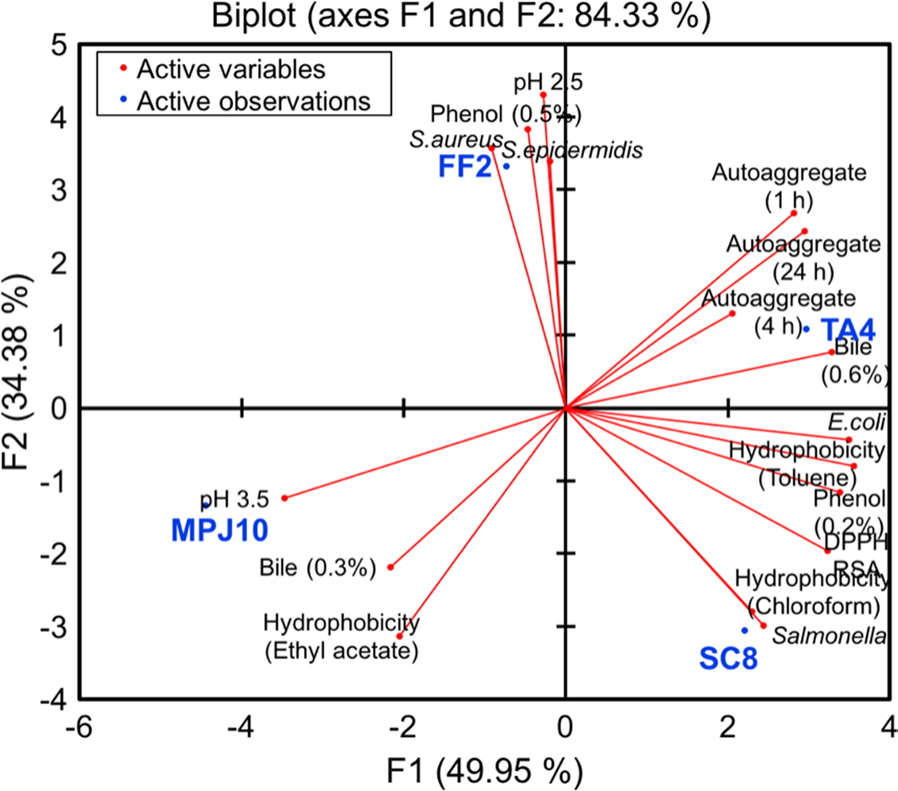
Principal component analysis (PCA) biplot projection based on probiotic attributes for selection of potential probiotic LAB strains. The principal components explain 84.33% of the total variance

Principal component analysis (PCA) was conducted to identify the promising LAB probiotic by investigating the correlation amongst the probiotic attributes, which were pH, bile, phenol tolerance, cell surface hydrophobicity, cellular autoaggregation, antimicrobial activity, and antioxidant activity (Fig. 5). PCA is designated with two principal components where the first principal component (F1) represents the maximum variation in the data and the second principal component (F2) covers the remaining variation to the first. Based on the analysis, the two principal components (F1 and F2) explained 84.33% of the total variation, while F1 and F2 accounted for 49.95% and 34.38% variance in the data, respectively (Fig. 5). The PCA revealed that the factorial space can be classified into four major groups in which the first group consists of strain TA4, which is located in quadrant I (positive side of both F1 and F2) and expressed the highest value for autoaggregation and bile tolerance attributes. The second group consists of strain FF2 located in quadrant II (positive side of F2 and negative side of F1), which exhibited high values for pH, phenol tolerance, and antimicrobial activity. The third group consists of strain MPJ10 located at quadrant III (negative side for both F1 and F2) and expressed high values for pH, bile, and hydrophobicity. The fourth group consists of strain SC8 located at quadrant IV (positive side of F1 and the negative side of F2), which expressed high values for phenol tolerance, antioxidant and antimicrobial activity, and hydrophobicity. In general, strains TA4 and SC8 possessed the highest levels of probiotic properties. In particular, strain TA4 showed the highest correlation with respect to variables with maximum factor scores (Table 5), clearly proving it as a probiotic candidate with the highest potential. Thus, strain TA4 was selected as a zinc-tolerant probiotic for microbial synthesis of ZnO NPs.

**Table 5:** Factor score of principal component (F1–F3) for potential probiotic strains

Observation	F1	F2	F3
TA4	2.967	1.080	2.168
SC8	2.208	− 3.059	− 1.483
FF2	− 0.730	3.324	− 1.670
MPJ10	− 4.445	− 1.344	0.985

#### Sequence analysis of the 16S rRNA gene

**Fig. 6:**
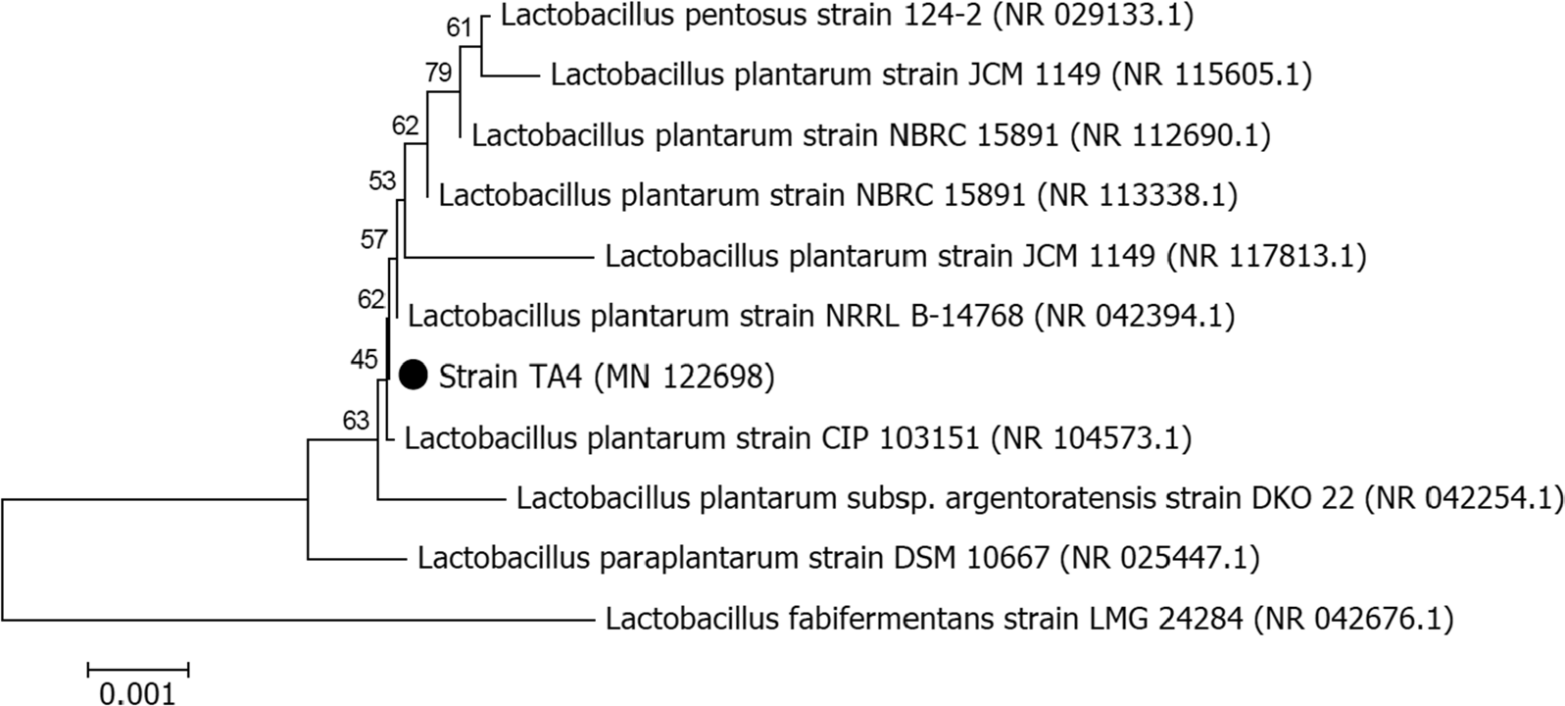
Phylogenetic tree of *L. plantarum* strain TA4. The tree is drawn to scale, with branch lengths in the same units as those of the evolutionary distances used to infer the phylogenetic tree. The analysis involved 11 nucleotide sequences

16S rRNA gene analysis of strain TA4 (1489 bp) revealed the highest degree of similarities to *Lactobacillus plantarum* CIP 103151 (100%), followed by *L. pentosus* 124-2 (100%), *L. plantarum* JCM 1149 (99.87%), *L. paraplantarum* DSM 10667 (99.8%), and *L. plantarum* NBRC 15891 (99.93%). Based on the phylogenetic relationship analysis using the neighbor-joining method (Fig. 6), strain TA4 was placed next to *L. plantarum*. Moreover, strain TA4 is Gram-positive and rod-shaped and form circular and creamy white colonies on MRS agar. Morphological and microscopic observation of strain TA4 together with the phylogenetic analysis revealed that the strain TA4 was tentatively identified as *L. plantarum* strain TA4. The 16S rRNA sequence of this strain has been deposited to the GenBank under the Accession number of MN122698.

### Characterization of biosynthesized ZnO NPs

#### UV–vis spectroscopy and dynamic light scattering (DLS)

**Fig. 7:**
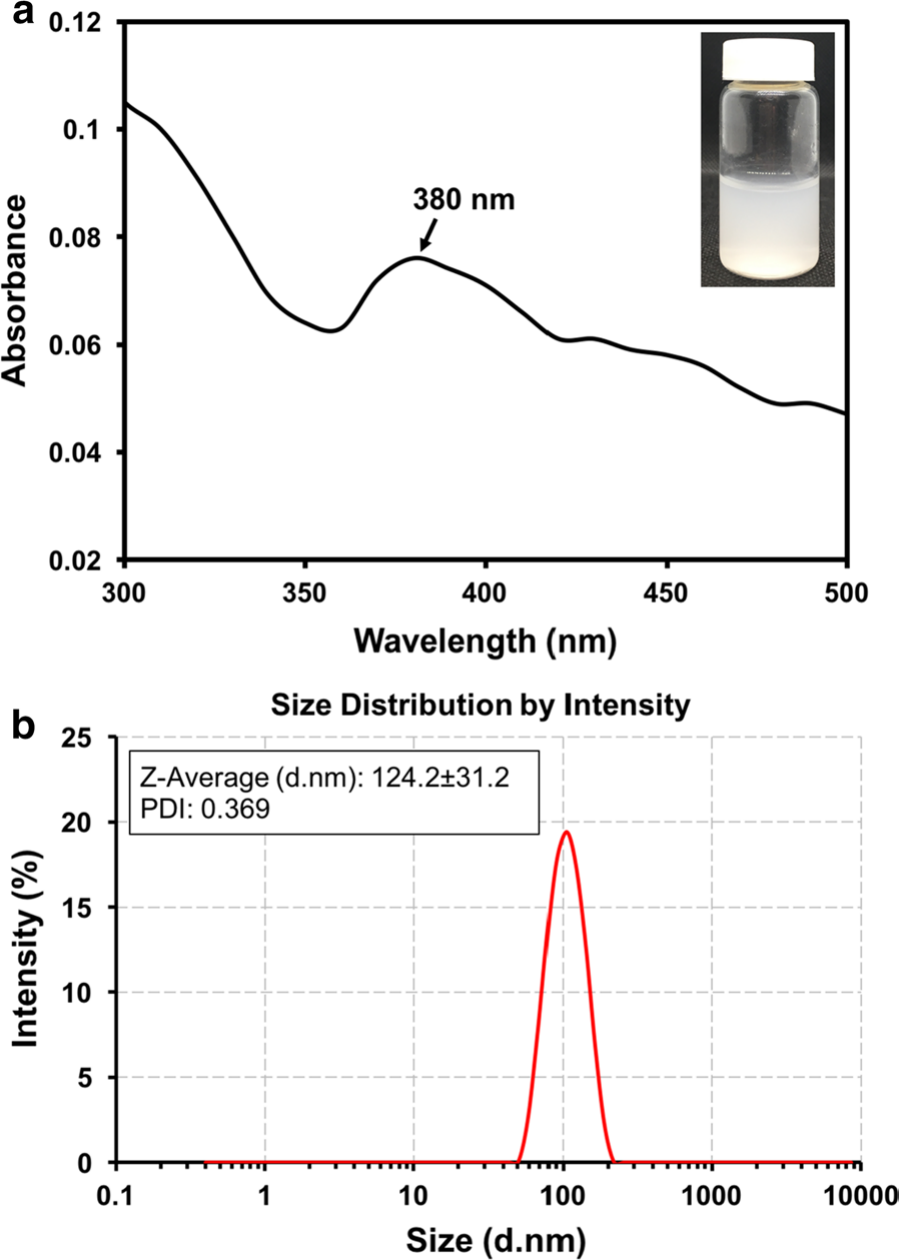
UV–vis absorption spectrum of biosynthesized ZnO NPs by *L. plantarum* strain TA4 cell biomass exposed to Zn^2+^. **a** Particle size distribution as obtained from DLS of biosynthesized ZnO NPs by *L. plantarum* strain TA4 (**b**)

The optical properties of ZnO NPs are essential aspects of the structure and feature characterization. The absorption peak of biosynthesized ZnO NPs showed maximum surface plasmon resonance (SPR) bands at the wavelength of 380 nm (Fig. 7a), which confirmed the formation of ZnO NPs. One of the standard methods to measure the average diameter of NPs in a colloid solution is by using dynamic light scattering (DLS). Laser diffraction revealed that the average hydrodynamic size of NPs obtained was 124.2 ± 31.2 nm along with the polydispersity index (PDI) of 0.369 (Fig. 7b), indicating monodispersed NPs.

#### Scanning electron microscope (SEM) and energy dispersive X-ray (EDX) analysis

**Fig. 8:**
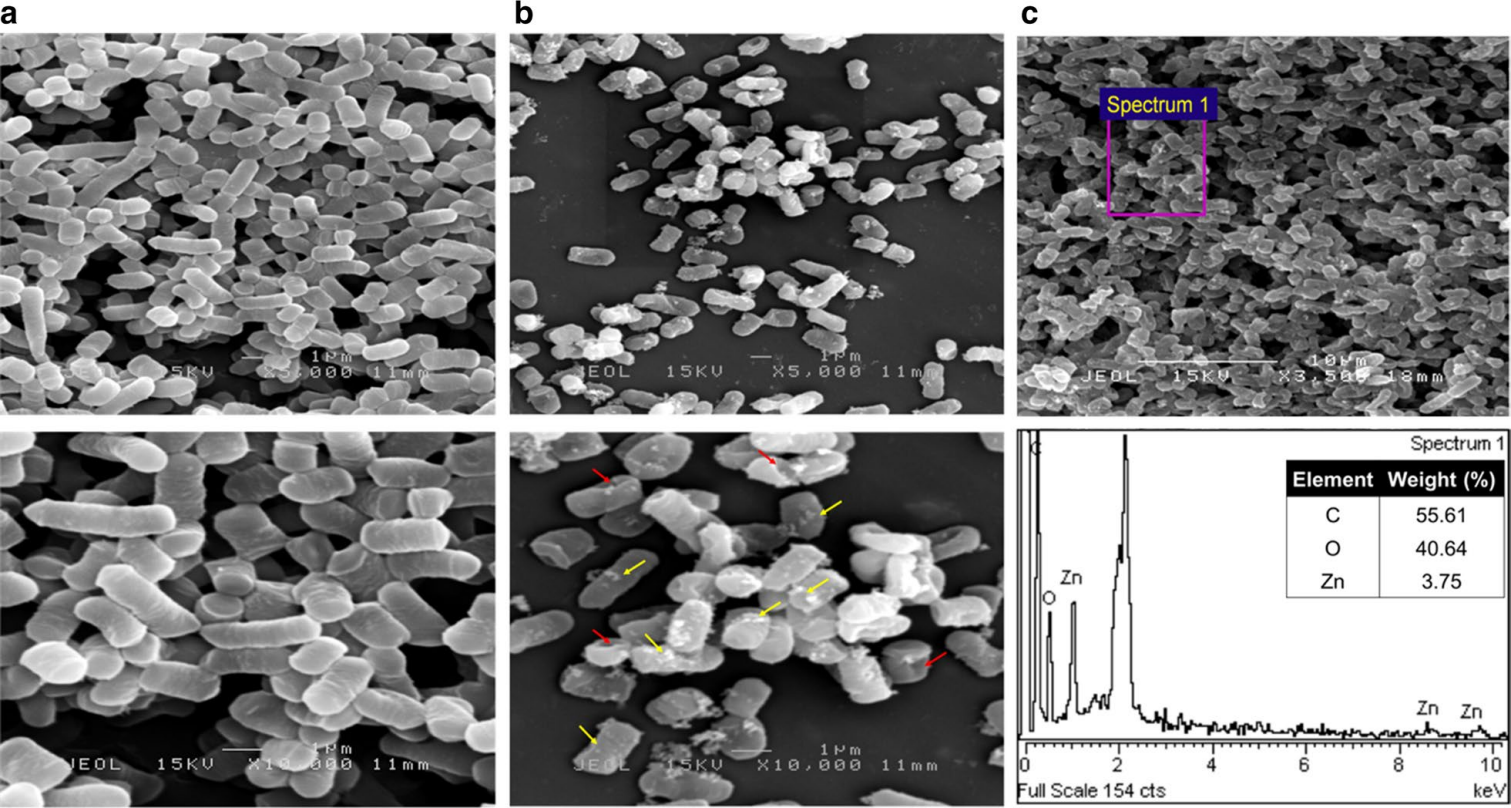
SEM micrographs of *L. plantarum* strain TA4. Control group (unexposed to Zn^2+^) (**a**), Treatment group (exposed to Zn^2+^) (**b**), EDX spectra of *L. plantarum* strain TA4 exposed to Zn^2+^ (**c**). Electron-dense NPs are located extracellularly as indicated by yellow arrow

To study the effect of Zn^2+^ exposure on cellular morphology of TA4 strain, scanning electron microscope (SEM) was performed. The micrographs obtained showed different changes in cell morphology of TA4 strain. The cells of the control group (Fig. 8a) exhibited smooth-surface and typically healthy rod-shaped of TA4 strain. In contrast, under the exposure of Zn^2+^, significant alteration in terms of cell membrane conformation is observed compared to the control, where the TA4 strain is no longer smooth, and there is a presence of perforated structure (red arrow) on the membrane surface (Fig. 8b). Such alterations on the cell membrane surface are presumably caused by the involvement of cell membrane in the biosorption process of Zn^2+^. Moreover, several particles are present on the membrane surface (yellow arrow, Fig. 8b), indicating the formation of biosynthesized ZnO NPs. Furthermore, the existence of Zn absorption peak of energy dispersive X-Ray (EDX) spectrum confirms the presence of elemental Zn. Moreover, the presence of other component peaks in the spectra is due to the chemical used for sample processing and gold biofilm during the coating process.

#### Fourier-transform infrared (FT-IR) spectroscopy analysis

**Fig. 9:**
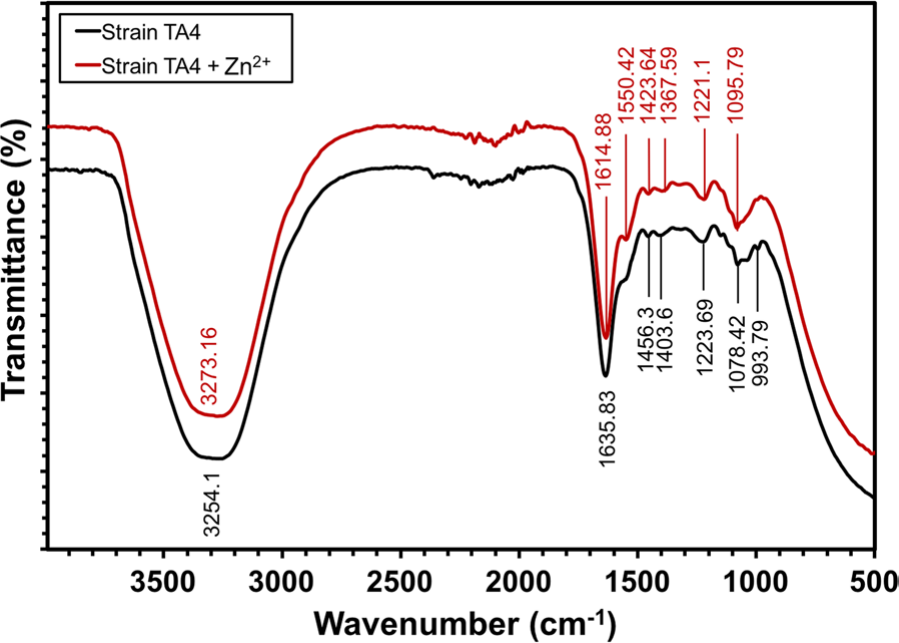
Comparison of FT-IR spectral analysis of *L. plantarum* strain TA4 (c). The black vibrational lines indicate the control of cell biomass (unexposed to Zn^2+^) and the red line indicate the cell biomass exposed to Zn^2+^

Fourier-transform infrared (FT-IR) analysis was performed to characterize and identify the difference between functional groups of the control sample (strain TA4 without Zn^2+^ exposure) and zinc-exposed cell biomass that was responsible for Zn^2+^ biosorption. Figure 9 represents the comparative FT-IR spectra of both samples. The control sample demonstrated the presence of six major absorption peaks at 3254.1, 1635.83, 1456.3, 1403.6, 1223.69, 1078.42, and 993.79 cm^−1^, which shows their typical complex nature characteristics. Meanwhile, the absorption peaks of the cells exposed to Zn^2+^ shifted to 3273.16, 1614.88, 1423.64, 1367.59, 1221.1, and 1095.79 cm^−1^. Additionally, there was an absorption peak at 1550.42 cm^−1^ that was not seen in the control. Table 6 represents the absorption peaks of strain TA4 exposed to Zn^2+^ and the interpretation of vibrational assignment with functional groups. The shifted in FT-IR spectra at 3273.16 cm^−1^ representing N–H asymmetric stretching of the amine and O–H bond of hydroxyl groups on the cell surface; 1614.88 cm^−1^ (C=O stretching) and 1550.42 cm^−1^ (N–H bending) attributed to amide I and amide II in protein, respectively; 1423.64 cm^−1^ indicates CH_3_ bending in proteins peptide bonds; while 1367.59 cm^−1^ and 1221.1 cm^−1^ indicate O–H bending and C–O stretching bands of the carboxylate ion group (COO^−^ symmetric stretching) with phosphate groups (P=O and P–O of the C–PO_3_^−2^) and 1095.79 cm^−1^ which corresponding to (C–C=O, C–O–P) phosphoprotein and hydroxyl groups from saccharides.

**Table 6:** Main absorption peaks and vibrational assignment with functional groups interpretation of *L. plantarum* strain TA4 exposed with Zn^2+^

Absorption peak (cm^−1^)	Vibrational assignment	Functional group	References
3273.16	O–H stretching and N–H asymmetric stretching	Amine and hydroxyl	[75]
1614.88	C=O stretching	Amide I	[45]
1550.42	N–H bending	Amide II	[45]
1423.64	CH_2_/CH_3_ bending	Lipids and proteins peptide	[76]
1367.59	C=O of COO^−^ symmetric stretching	Carboxylate	[19]
1221.1	P=O. P–O	Phosphate	[45]
1095.79	C–C=O, C–O–P	Hydroxyl from saccharides, phosphoprotein	[74, 77]

## Discussion

In this study, a zinc-tolerant *L. plantarum* strain TA4 was isolated from locally fermented food (*tapai pulut*) that demonstrates probiotic properties. In fact, the previous study reported the presence of LAB with probiotic potential from *tapai pulut*, which is identified as *L. fermentum* [28]. In particular, *Lactobacillus* is a Gram-positive, rod-shaped bacteria that is known to predominate probiotics. Notably, its presence maintains the microflora in the gut ecosystem and provides health benefits [10]. Besides that, most *lactobacilli* species are generally regarded as safe (GRAS) and deemed to be non-pathogenic because they are not associated with any diseases. Probiotic attributes are determined by several factors; acid and bile tolerance, hydrophobicity and aggregation ability, as well as health-promoting (antimicrobial and antioxidant) benefits [10]. As the probiotic bacteria pass through GIT, they encounter harsh conditions such as acidic and bile salts (stresses conditions) before they can colonize the small intestine. As shown in Table 2, most of the LAB strains (after 4 h of exposure) in this study demonstrated high survival rate under the following conditions, specifically at pH 2.5 and pH 3.5 and bile concentrations of 0.3% and 0.6%. These results indicate the strain robustness in resisting the challenge, which was in line with the reported results of a prior study by Giri et al. [29] where *L. plantarum* L7 were found to survive and retain their viability under similar conditions, specifically at pH 2 and pH 3 and bile concentration of 0.3%. Besides that, all LAB strains in this study demonstrated tolerance towards phenol at bile concentrations of 0.2% and 0.5%. The growth of bacterium was also found unaffected after 24 h, which demonstrated the ability of this strain to withstand the phenolic stress conditions. Phenol is known as a toxic metabolite that is produced from deamination of various amino acids by gut microorganisms derived from dietary based-protein, which exerts bacteriostatic effects on gut microbiota [30].

The probiotic adherence ability on epithelial cells is important for health-promoting effects, which is required to maintain their intestinal colonization, besides facilitating the augmentation of intestinal mucosal barrier. Therefore, this will prevent cells from the adherence of pathogenic bacteria [31], thus increase the immunity of the organism. It was reported that the hydrophobicity and autoaggregation ability of the probiotic is associated with adherence ability, which is used as an indicator to evaluate bacterial adhesion ability [32]. The obtained results revealed that the LAB strains showed varying degrees of hydrophobicity to toluene, which ranged from 11.4 to 58.0%. In particular, the TA4 strain recorded the highest value. Hydrophobicity of above 50% is deemed acceptable for probiotics. Rondon et al. [33] reported similar results where *L. salivarius* strain C65 isolated from broiler chicken recorded hydrophobicity of 55.03%. The variation in cell hydrophobicity occurs due to the presence of different cell wall constituents, such as phosphate, carboxylate groups, and proteins, which influence the surface hydrophobicity [32]. Additionally, high autoaggregation ability of TA4 strain after incubation for 4 h in this study increased throughout the 24-h period, which was also observed in other strains. Angmo et al. [34] reported a similar trend where the autoaggregation activity of *L. plantarum* strains in the study increased as the incubation time increased. It is plausible due to the presence of proteins and polysaccharides on the cell surfaces that enable them to aggregate [35].

Meanwhile, the non-neutralized cell-free supernatant (CFS) of TA4 strain demonstrated antagonistic activity against the tested pathogens. However, the neutralized CFS did not show any inhibitory activity against the same pathogens. These results demonstrated the influence of the organic acid produced (based on the final pH) on the inhibitory activity and that this strain is not a bacteriocin producer. Similarly, Hwanhlem et al. [36] reported no presence of bacteriocin-producing strains and that the pH reduction by organic acids led to the antibacterial activity of isolated *L. plantarum* from fermented shrimp (*Kung*-*Som*). The antimicrobial effects of LAB are attributed to the production of inhibitory substances, such as organic acids (such as lactic, propionic, acetic and succinic acids), hydrogen peroxide, and bacteriocins [37].

Accordingly, an excessive production of reactive oxygen species (ROS) causes cellular oxidative damage to a human body. The mechanism of probiotic antioxidant ability may be poorly understood; indeed, the antioxidant potential manifests through the chelation of metal ions, production of antioxidase, and enzyme suppression for the production of ROS [38]. DPPH is typically used to measure the antioxidant activity as it is a stable organic radical compound. In this study, the CFS of TA4 strain demonstrated greater DPPH scavenging activity at 80.5%, which reaffirmed their antioxidant capability; thus, indicating their health-promoting benefits. This observation is consistent with the study of Riaz Rajoka et al. [39] that reported higher antioxidant activity of CFS of several *L. rhamnosus* strains, ranging from 84.0 to 88.0%. Adding to that, the ability of TA4 strain to withstand metal ion in this study also explains its high antioxidant ability. Certain bacteria can adapt with high metal ion level via the formation of antioxidant enzyme superoxide dismutase (SOD) [40] that protects them from metal ion toxicity. For instance, the presence of high antioxidant enzyme when *S. aureus* was exposed to Zn^2+^ was said to facilitate the ability of the bacteria to cope with oxidative stress caused by metal ions [41]. Moreover, the adhesion to both chloroform (acidic, electron acceptor) and ethyl acetate (basic, electron donor) were also tested in this study to assess the cell surface Lewis acid–base characteristics of bacteria. In particular, the TA4 strain recorded high chloroform affinity at 54.9%, which indicates the basic (electron donor) property of TA4 strain. The electron donor property of TA4 strain implies the strain electrostatic interactions to bind with metal cations that are associated with the presence of carboxylic (–COO^−^) and hydrogen sulfite (–HSO_3_^−^) functional groups on the bacterial surface [21, 42]. On the other hand, the lower affinity (41.4%) to ethyl acetate observed in this study indicates the non-acidic and poor electron acceptor properties of the TA4 strain.

Several *Lactobacillus* strains have been reported to resist various metals ion at different concentration levels. Most studies emphasize on bioremediation or decontamination of heavy metals in the body and environmental perspectives [19, 25, 42–48]. However, less is known about their capacity to tolerate with Zn^2+^ and produce zinc nanomaterials simultaneously. Zinc is an essential metal ion, however, at higher concentration, it is toxic to the bacteria [26] so bacteria evolve to protect themselves by reducing the ions to NPs. This shows that bacteria can tolerate high level of metal ions, thus act as a microbial nanofactory. Presentato et al. [49] proved that the isolated *Rhodococcus aetherivorans* BCP1 which have high tolerance toward selenium ion concentration at 500 mM are capable to produce selenium nanoparticles (Se NPs). In this study, based on the PCA results, strain TA4 was chosen as a potential probiotic which exhibited the capacity to tolerate higher Zn^2+^ concentration at 500 mM. The results indicate the presence of highly resistant LAB against Zn^2+^ as compared to the resistant LAB studied by Leonardi et al. [50], who reported the maximum tolerance concentration (MTC) against Zn^2+^ of several *Lactobacillus* and *Bifidobacterium* strains at 100 mM. Moreover, former studies reported that *Pseudomonas* sp. SN7 and SN28 demonstrated MTC against Zn^2+^ at 25 mM [51] whereas *Sphinomonas* sp. strain DX-T3-03 isolated from a copper mine tailing exhibited resistance to Zn^2+^ up to 40 mM [52]. To our knowledge, we have reported a zinc-tolerant probiotic with the highest MTC value of 500 mM compared to other reports in the literature. In fact, the variations in resistance against metal ions between bacterial species are due to different properties of the bacteria, which include cell wall structure, functional groups and surface area [20, 26, 46].

To validate the formation of ZnO NPs by the cell biomass of strain TA4, a UV–vis spectroscopy analysis was conducted. As a result, a notable absorbance peak was obtained at 380 nm from the UV–vis spectroscopy, proving the formation of ZnO NPs. This absorbance rate was due to the surface plasmon resonance (SPR) band of ZnO NPs. This finding was in an agreement with other studies which reported a similar absorption peak [53, 54]. According to Eltarahony et al. [54], it could be inferred that it was also due to the uniform particle size distribution. Notably, it was also proven in this DLS analysis study that the biosynthesized ZnO NPs by TA4 cells were monodispersed in nature due to the ideal PDI value obtained at 0.369.

In this study, DLS analysis found that the average size distribution produced by TA4 strain amounted to 124.2 ± 31.2 nm. The average size of biosynthesized ZnO NPs produced by strain TA4 was within the range of biosynthesized ZnO NPs obtained through *Rhodococcus pyridinivorans* NT2 [55]. However, this range of size was larger than the sizes produced in other studies. Specifically, the average size of ZnO NPs amounted to 7–19 nm through *L. plantarum* strain in Selvarajan and Mohanasrinivasan [12], and 57.72 nm average size was obtained through using *Aeromonas hydrophila* strain in Jayaseelan et al. [56]. Essentially, small particle size is one of the main parameters which determine the physicochemical of ZnO NPs associated with their high surface area to volume ratio. This characteristic distinguishes them from their bulk counterparts. Nonetheless, the particle size obtained from DLS was normally larger than the average size obtained from electron microscopy method due to the effect of Brownian motion [57]. This difference of size was also due to the hydrodynamic size, where measurements were conducted based on the size of the metal core and the biological compound bound on the particle surface. As a result, an increase in particle size occurred [58]. Therefore, it is recommended that future studies conduct particle size characterization through a transmission electron microscope (TEM).

Lactic acid bacteria created exciting microorganisms which naturally possess properties which function in either intracellularly or extracellularly reducing metal ions into their respective metal NPs [5]. However, there is insufficient understanding of the actual mechanisms involved in the formation of ZnO NPs through LAB, Therefore, further insights on this matter are essential. This study found that the cells exposed to Zn^2+^ displayed pronounced structural alterations, indicating the involvement of cell membrane in the biosorption process and their function as the template for ZnO NPs biosynthesis. Furthermore, the EDX analysis of the strain TA4 cell detected a presence of zinc elemental composition, indicating the successful biosynthesis of ZnO NPs. Overall, these findings were in an agreement with previous studies on the involvement of cell membrane in the biosynthesis of selenium NPs [59], tellurium NPs [60], and gold NPs [58] by other microorganisms.

Nevertheless, Bustos et al. [61] reported that the biosynthesized NPs on living cells were not only apparent on the cell surface, but they were also present on the surrounding. It was also inferred that NPs biosynthesis by the microorganism did not only occur through adsorption (biosorption) as it could also take place through the absorption (bioaccumulation) of the metal ions and formation of NPs inside the cell. Based on TEM observations, the formation of intracellular NPs was also found in *Ochrobactrum* sp. strain, where the particles were found inside the cell. However, no extracellular NP was detected in SEM micrographs [59]. In this study, the depositions of extracellular ZnO NPs on cell surfaces were detected (refer to Fig. 8b), implying the occurrence of ZnO NPs biosynthesis through extracellular biotransformation by cell membrane.

The bacterial cell wall is the first component which had contact with the metal ion. It plays an important role as the barrier and binding site for the metal ion. The cell wall of LAB consists of peptidoglycans, (lipo)teichoic acids, protein, and polysaccharides [16, 21]. Meanwhile, protein is the most abundant component of the *Lactobacillus* cell surface. It is also known as S-layer (glycol) proteins [62], which comprise numerous functional groups such as carboxyl, hydroxyl, amine, and phosphate. These functional groups were predicted to possess various ligands and charge distributions which bound the cationic ions of Zn^2+^ [19]. These findings were supported by the FT-IR analysis, which identified the functional groups present on the cells strain TA4 involved in the biosorption and biosynthesis processes of ZnO NPs. The role of the aforementioned functional groups in Zn^2+^ binding process was determined by observing the shifts in their absorption peaks.

Based on Table 6, it was found that the strain TA4 cells, which were exposed to Zn^2+^, showed some shifts on the absorption peaks of carboxylate (–COOH), hydroxyl (O–H), amine (N–H), and phosphate (P=O, P–O) functional groups compared to the control (unexposed to Zn^2+^) functional group. This finding indicated the essential role of –COOH, O–H, N–H, and P=O, P–O functional groups in Zn^2+^ binding process and the formation of ZnO NPs. These results were in an agreement with Król et al. [7] and Tripathi et al. [63], who found that the presence of peak corresponded to the carboxyl and amide groups on the ZnO nanocomposite and ZnO nanoflowers biosynthesised by *L. paracasei* and *Bacillus licheniformis* strain respectively. This biosynthesis involved the formation and stabilisation of ZnO NPs. In addition, the EPS secreted by *L. plantarum* was reported in Garmasheva et al.’s [15] study, where the reduction and biosorption of metal ion were involved, leading to the formation of NPs. However, this formation was contradicted with this study’s finding as the TA4 strain investigated in this study did not produce EPS. Therefore, it was proven that this TA4 strain was highly dependent on its cell wall components to detoxify metal ions, including reducing and developing NPs.

Overall, some of the probiotic features of TA4 strain play a vital role in ZnO NPs biosynthesis. ZnO NPs are mainly obtained through chemical and physical methods. However, these methods involve the use of harsh chemicals, where their application would potentially result in low biocompatibility and risk to living organisms [64]. Nevertheless, microbial or biological synthesis has gained significant attention in the synthesis of ZnO NPs due to its eco-friendly nature, biocompatibility, and the involvement of non-toxic chemicals [5]. Despite the advantages, the primary concern in using microorganism for the biosynthesis of ZnO NPs is their low yield productivity, which remains a challenge. To attain the maximum yield of NPs, it is necessary to optimize the cultural conditions and various physical parameters, including temperature, pH, precursor concentration, and reaction time [5]. It has been reported that these optimization parameters influence the yield productivity of NPs [65, 66]; therefore, further investigation is required. Furthermore, it was found in this study that strain TA4 had the potential to function as a nanofactory, ensuring a sustainable approach for ZnO NPs production. It also possessed high resistance to Zn^2+^ as it was equipped with probiotic properties. Due to these attributes, strain TA4 possessed an advantage in its biotechnological application in the bioremediation, food, and pharmaceutical industry. Moreover, its ability to bind Zn^2+^ would be useful in preventing excessive dietary Zn^2+^ toxicity in animals and human gut [67] and in the removal of high concentration of Zn^2+^ from consumption of water and liquid food [20]. Notably, zinc-enriched LAB was reported to possess beneficial effects on living organisms [50]. This positive discovery would contribute to a new perspective regarding zinc-enriched LAB use as an organic matrix for a zinc dietary supplementation strategy for human and animals [20].

## Conclusion

In conclusion, a sustainable approach for the production of ZnO NPs was developed using zinc-tolerant probiotic *L. plantarum* strain TA4, which was isolated from local fermented food. This strain was shown to possess probiotic characteristics and the strongest resistance to a high Zn^2+^ concentration. This was followed by the transformation of Zn^2+^ into ZnO NPs. It was proven from the SEM–EDX and FT-IR analysis used in this study that the biosynthesis of ZnO NPs occurred through biosorption of Zn^2+^ in the presence of functional groups on the cell membrane of TA4 strain. These functional groups functioned as the ligands by attracting the Zn^2+^ through electrostatic interaction. The Zn^2+^ was then reduced to Zn^0^ prior to its conversion to ZnO NPs. Furthermore, the zinc-tolerant together and the probiotic properties of strain TA4 contributed to a new possibility for the future decontamination of Zn^2+^ and dietary strategies. It was also indicated that an environmentally sustainable, cost-effective, and biocompatible microbial cell nanofactory is essential for ZnO NPs production. Finally, it is recommended for future studies to characterize the physicochemical properties of the biosynthesised ZnO NPs production through strain TA4 and evaluate their properties for biological application.

## Materials and method

### Preparation of zinc solution

A 1 M stock solution of Zn^2+^ was prepared by dissolving zinc sulfate (ZnSO_4_∙7H_2_O) in deionized water. The Zn^2+^ solution was sterilized by filtration and added to the bacterial culture medium.

### Isolation of lactic acid bacteria (LAB)

Lactic acid bacteria was isolated from local fermented food, fruit peels, and silage using de Man, Rogosa, and Sharpe (MRS) medium. Each of the sample sources was enriched in 250 mL Erlenmeyer flasks containing MRS broth and incubated for 24 h at 37 °C with 150 rpm agitation. After the incubation, the bacterial culture was serially diluted, plated on MRS agar, and incubated for 24 h at 37 °C. Single colonies showing different morphologies were obtained by streak plate technique. The cell morphology, Gram-stain property, and catalase reaction of all isolates were observed and recorded. Gram-positive and catalase-negative isolates were selected and stored at − 80 °C in MRS broth containing 25% (v/v) glycerol for further processing.

### Determination of zinc-tolerant LAB and maximum tolerable concentration (MTC)

Zinc-tolerant LAB was screened by inoculating the LAB strains in MRS medium containing 10 mM Zn^2+^ and incubated at 37 °C for 24 h. LAB strains showing growth were selected for subsequent studies. To determine the MTC of LAB strains against Zn^2+^, the agar well diffusion method was carried out according to Hassen et al. [68] methods. Zn^2+^ solutions were prepared in different concentrations (20, 40, 60, 80, 100, 200, 300, 400, 500, and 600 mM). Sterile MRS agar plates were prepared and each plate was spread with overnight cultures of LAB strains. Wells were punched by a sterile borer with 6 mm in diameter and 100 µL of Zn^2+^ solution of each concentration was added to each well and incubated at 37 °C for 24 h. After incubation, the inhibition zones were recorded by measuring the distance of the edge of the zone to the edge of the well. LAB strains showing no clear zone and zone size of 1 mm or less were considered as resistant strains [69]. The LAB strains that showed the highest MTC value were selected for further studies. Test tube method was performed to ascertain the survivability of the strains in broth medium at different Zn^2+^ concentrations. The strains exhibiting the highest MTC value were grown in MRS broth containing different Zn^2+^ concentrations. A stock solution of Zn^2+^ was filter-sterilized and added in an appropriate amount with the concentration ranging from 20 to 500 mM. The culture was incubated at 37 °C for 24 h and the number of cells is recorded as the average of the colony-forming unit (log CFU/mL) for each of the test tube. The experiment was performed in triplicate and mean values and standard deviations were calculated.

### In vitro probiotic characterization of zinc-tolerant LAB

The characterization of potential probiotic LAB was screened using the following methods.

### Determination of pH, bile, and phenol tolerance

Acid and bile salt tolerance were determined according to the method describe by Ji et al. [70] with some modifications. About 1 mL of the overnight grown strains were transferred to 9 mL of fresh MRS medium adjusted to pH 2.5 and 3.5 with HCl and incubated at 37 °C for 4 h. Similarly, for bile tolerance, the strains were transferred to MRS broth containing 0.3% and 0.6% oxgall (Oxoid, Basingstoke, UK) and incubated at 37 °C for 4 h. The number of viable cells was determined at 0 and 4 h of incubation time by serially diluting the culture before plating it on MRS agar. Survival rates were determined by the number of viable cells present on the agar and calculated as colony-forming unit (CFU) per mL of sample. The survival percentage was calculated as follows:

$$ Survival\;rate \; \left( \% \right) = \frac{{Number\;of\;viable\;cells\;survived \left( {{\text{CFU}}/{\text{mL}}} \right)}}{{Number\;of\;initial\;viable\; cells\;inoculated \left( {{\text{CFU}}/{\text{mL}}} \right)}} \times 100 $$

Phenol tolerance was determined by inoculating the strains in 10 mL MRS broth containing 0.2% and 0.5% phenol (v/v). Strains inoculated in MRS broth without phenol were used as controls. The cultures were incubated at 37 °C for 24 h and viable cells were counted (CFU/mL). All experiments were performed in triplicate and the mean values and standard deviations of each sample were calculated.

### Determination of cell surface hydrophobicity, cellular autoaggregation and exopolysaccharide (EPS) production

The hydrophobicity of the strains was evaluated by microbial adhesion to solvents (MATS) method according to Rosenberg et al. [71]. Overnight cultures were harvested by centrifugation at 5000 rpm for 10 min and washed twice with PBS (pH 7.4). The harvested pellets were resuspended in the same PBS to an absorbance of 0.8 at 600 nm (A_0_). Equal volumes (1:1) of solvent and suspension were mixed and vortexed thoroughly for 2 min. The mixture was incubated at 37 °C for 30 min for phase separation and the aqueous phase was spectrophotometrically measured (A_1_) at 600 nm. Three solvents were used in this study, which were toluene (an apolar solvent), chloroform (monopolar and acidic solvent), and ethyl acetate (monopolar and basic solvent). The percentage of hydrophobicity was calculated as follows:

$$ Hydrophobicity \;\left( \% \right) = \frac{{\left[ {Initial\;optical\;density \; \left( {A_{0} } \right) - Final\;optical\;density \; \left( {A_{1} } \right)} \right]}}{{Initial\;optical\;density \; \left( {A_{0} } \right)}}  \times 100 $$

The autoaggregation of the strains was measured by using methods described by Giri et al. [29]. Overnight cultures were centrifuged at 5000 rpm for 10 min and the pellets were washed repeatedly with PBS and resuspended in PBS (pH 7.4) to an absorbance 1.0 at 600 nm (A_0_), vortexed for 30 s, and incubated at 37 °C in static condition. The OD of the suspension (1 mL) was measured at 1 (A_1_), 4 (A_1_), and 24 h (A_1_) at 600 nm. The percentage of autoaggregation was calculated as follows:

$$ Autoaggregation \; \left( \% \right) = \frac{{\left[ {Initial\;optical\;density\; \left( {A_{0} } \right) - Final\;optical\;density\; \left( {A_{1} } \right)} \right]}}{{Initial\;optical\;density \;\left( {A_{0} } \right)}} \times 100 $$

The screening process for EPS production was performed according to Nambiar et al. [72] method. Briefly, each of the strain was grown on MRS agar supplemented with 5% glucose (w/v) and incubated at 37 °C for 24–48 h. The EPS producing-strains was identified by loop touch method to observes the ropy or mucoid features of the strains. All experiments were performed in triplicate and the mean values and standard deviations of each sample were calculated.

### DPPH free RSA

The antioxidant activity (free radical-scavenging) of the LAB strains was determined according to Son and Lewis’s [73] method. Overnight LAB strains were harvested by centrifugation at 5000 rpm for 10 min and cell-free supernatant (CFS) were collected. Two hundred microliters of freshly prepared DPPH (6 mg/100 mL methanol) was added with an equal volume of methanol-containing CFS (10 µL CFS + 190 µL methanol) and thoroughly vortexed. The mixture was incubated at room temperature in the dark for 30 min. A mixture of DPPH with methanol (1:1 volume ratio) was prepared as a control whereas methanol was used as a blank. After the incubation, the absorbance was measured at 517 nm using a UV spectrophotometer and the assay was performed in triplicate. The percentage of DPPH radical scavenging activity was calculated as follows:

$$ RSA_{{\left( {DPPH} \right)}} \left( \% \right) = \frac{{\left( {A_{control} - A_{test} } \right)}}{{A_{control} }} \times 100 $$

### Antibacterial activity

The antibacterial activity of the cell-free supernatant (CFS) of LAB strains was assessed against Gram-positive and Gram-negative pathogens by agar well diffusion method. Non-neutralized CFS were prepared from overnight cultures by centrifugation (5000 rpm) and filtered, while neutralized CFS were prepared by adjusting to pH 6.5 to 7.0 with 1 M NaOH followed by filtration. The efficacy of antibacterial activity using non-neutralized and neutralized CFS was measured by observing the inhibition zones around the well. Clear zone indicates a positive result and is expressed in millimeters. The test pathogens used were *Staphylococcus aureus*, *Salmonella* sp., *Escherichia coli*, and *Staphylococcus epidermidis*. The test was performed in triplicate.

### Molecular identification of zinc-tolerant probiotic LAB

The selected strain was subjected to molecular identification. Genomic DNA was extracted using QIAamp DNA Mini Kit (Qiagen GmbH, Hilden, Germany) according to the manufacturer’s instruction and amplified using universal primers 27F (5′-AGAGTTTGATCCTGGCTCAG-3′) and 1492R (5′-GGTTACCTTGTTACGACTT-3′). The PCR product was purified using the QIAquick PCR Purification Kit (Qiagen, Germany) and sent for sequencing at First BASE Asia Sdn. Bhd. (Malaysia). The resulting sequence of the strain was then compared using the NCBI Basic Local Alignment Search Tool (BLAST; http://www.ncbi.nlm.nih.gov/) to search for a similar sequence in GenBank. Sequences and their closest relatives were retrieved and aligned with ClustalW. A phylogenetic tree for the nucleotide sequences was constructed using the neighbor-joining method by using MEGA software version 7 (The Biodesign Institute, Tempe, AZ, USA). Evolutionary distances of nucleotide sequences were computed using the Jukes–Cantor model (bootstrap values: 1000 resampling).

### Biosynthesis and extraction of ZnO NPs

The biosynthesis of ZnO NPs was performed according to Markus et al. [58] method with minor modification. Briefly, *L. plantarum* strain TA4 was grown in MRS broth and incubated at 37 °C for 24 h with 150 rpm agitation. After the incubation period, the cells were recovered by centrifugation (5000 rpm, 20 °C) for 15 min and washed three times with PBS buffer. The cell biomass was then suspended in sterilized deionized water containing Zn^2+^ at concentrations of 500 mM and incubated at 37 °C for 24 h. After incubation, cells were collected by centrifugation at 5000 rpm for 10 min and washed with saline before being suspended in buffer. To obtain the ZnO NPs, cells were disrupted by alternating cycles of ultrasonication at 100 W for 5 min and continuous centrifugation at 5000 rpm for 5 min. Afterwards, the ZnO NPs were collected by high-speed centrifugation at 13,000 rpm for 15 min and washed with 80% ethanol to remove any undesirable components. The ZnO NPs produced were collected and air-dried overnight at 60 °C.

### Characterization of biosynthesized ZnO NPs

#### UV–vis spectra and DLS analysis

The formation of biosynthesized ZnO NPs by strain TA4 was monitored by visual assessment using UV–vis spectroscopy (Uviline 9400, Secomam, France) in a colloid solution. The spectrum was measured by observing the intense absorbance peak related to surface plasmon excitation in the wavelength range of 300–500 nm operated at a resolution of 1 nm. The hydrodynamic particle size distribution and polydispersity index (PDI) of biosynthesized ZnO NPs in solution was measured by DLS using Nano S (Malvern Instruments, UK). Briefly, about 100 µL of ZnO NPs solution was diluted in deionized water and vortexed for homogenization before performing the measurement [74]. The measurements were carried out in triplicate, and the data obtained was in the average value generated by the software equipped with the DLS instrument. The results were presented at intensity (%) and size number-based distributions.

#### SEM and EDX analysis

The morphology and elemental composition of TA4 strain exposed to Zn^2+^ were analyzed through SEM and EDX. The TA4 strain was grown overnight in MRS broth and incubated at 37 °C with 150 rpm agitation. Then, cell biomass was collected by centrifugation (5000 rpm, 10 min) and washed twice with PBS buffer. The cell biomass was then suspended in 100 mL of sterilized deionized water containing Zn^2+^ at the concentration of 500 mM in 250 mL flask and incubated at 37 °C for 24 h. After the incubation, the cells were recovered by centrifugation (5000 rpm, 10 min, 4 °C) and were subjected to sample processing for SEM. Briefly, the samples were fixed in 2.5% glutaraldehyde for 4 h at 4 °C and washed thrice with 0.1 M sodium cacodylate buffer. Afterwards, the samples were post-fixed with 1% osmium tetroxide for 2 h at 4 °C and washed again with the buffer for three times. The samples were dehydrated with graded acetone series (35, 50, 75, 95, and 100%, 10 min per step) and were then critical point-dried before mounted on the stub and subsequently coated with gold film in the sputter coater. SEM observation was performed using JEOL JSM-6400. The micrographs images were recorded and processed with Adobe Photoshop. Elemental analysis of strain TA4 cells was conducted using EDX, which was carried out using SEM equipped with an EDX spectrometer.

#### FT-IR spectroscopy analysis

The role of functional groups in Zn^2+^ binding on the bacterial cell was determined by FT-IR spectra analysis according to Mrvčić et al. [19]. Briefly, the bacterial cells of strain TA4 exposed to Zn^2+^ for 24 h were prepared and centrifuged at 5000 rpm for 20 min after the incubation period. The cells were washed twice with Milli-Q water and dried overnight. Bacterial cells without Zn^2+^ exposure were prepared as control. The spectra were determined in the region of 4000–500 cm^−1^ at a resolution of 4 cm^−1^ using Nicolet 6700 (Thermo Scientific).

#### Statistical analysis

Data from each experiment were analyzed by one-way analysis of variance (ANOVA) and the comparison between the treatments was performed using Tukey’s test with a significance level of *p* < 0.05. All the statistical analyses were performed using OriginPro software (version 9; OriginLab Corporation, Northampton, MA, USA). Statistical differences among the LAB strains were analyzed through Principal component analysis (PCA) using XLSTAT_TM_ software (Addinsoft, Paris, France). Four strains (TA4, SC8, FF2, and MPJ10) were analyzed where the discriminating variables were acid tolerance, bile tolerance, phenol tolerance, cell surface hydrophobicity (chloroform, toluene, ethyl acetate), cellular autoaggregation (1, 4, 24 h), antioxidant activity (DPPH RSA), and antimicrobial activity (*E. coli*, *Salmonella* sp., *S. aureus*, *S. epidermidis*).

## Data Availability

The datasets used and/or analyzed during the current study are available from the corresponding author on reasonable request.

## Abbreviations

LAB: lactic acid bacteria
ZnO NPs: zinc oxide nanoparticles
Zn: zinc
MTC: maximum tolerable concentration
DPPH: 2,2-diphenyl-1-picrylhydrazyl
RSA: radical scavenging activity
PCA: principal component analysis
DLS: dynamic light scattering
PDI: polydispersity index
SEM: scanning electron microscope
EDX: energy dispersive X-ray
FT-IR: Fourier transform infrared
TEM: transmission electron microscope
CFS: cell-free supernatant
